# Editorial: The unlearning of school attendance: ideas for change

**DOI:** 10.3389/fpsyg.2024.1401168

**Published:** 2024-04-02

**Authors:** Carolyn Gentle-Genitty, Arya Ansari, Michael Gottfried, Ineke Marshall

**Affiliations:** ^1^School of Social Work, Indiana University Bloomington, Bloomington, IN, United States; ^2^College of Education and Human Ecology, The Ohio State University, Columbus, OH, United States; ^3^Graduate School of Education: Policy, Organizations, Leadership, and Systems Division, University of Pennsylvania, Philadelphia, PA, United States; ^4^School of Criminology and Criminal Justice, Northeastern University, Boston, MA, United States

**Keywords:** unlearning, MTSS, attendance, absenteeism, health conditions, future studies, missing school

## Introduction

Unlearning attendance is an interrogation of the field and proposed blueprint for future studies on attendance and absenteeism.

Since the invention of schools (Elliott et al., 1993), work on attendance has centered on benefits and harm (Ceci and Williams, 1997), both within and outside of educational settings (Hanushek et al., 2008). The focus has largely been on understanding the links between attendance and learning outcomes (Elliott et al., 1993) as opposed to the people, their health conditions, their racial and ethnic disparities, interventions across school types, and the data for reconfigurations at systemic and analytic levels.

Despite existing efforts, high rates of absenteeism continue to influence adolescents (LeBoeuf et al.) and concerns have been raised among preschool students as well (Purtell and Ansari). We may learn a lot from Kearney et al.'s primer on the field's past and future trajectory to help us to evaluate our present.

We must critically reflect on our own practices if we are to be knowledge stewards and carers of our students, our schools, and those working within and around them. Using an umbrella systematic review (Jay et al.), machine learning (Bowen et al.), quasi-experimental evaluation (Arbour et al.), and conceptual and trend analysis, 44 researchers involved in this Research Topic assessed the field's current status. The scholars pointed to glaring reasons for a needed reflective pause. They examined the status of students from preschool to college (Korotchenko and Dobbs) and from early childhood (Purtell and Ansari) to adulthood and shared a threaded story of what is happening and why we are now at a critical juncture (Heyne et al.).

Our Frontiers special topic aimed to provide a “pause” to allow for critical assessment of current knowledge in the field of attendance and absenteeism, including ideas for change from the world's leading experts and trailblazers. These 44 scholars studying in 21 fields of study, ranging from Education, Psychology, Social Work, and Sociology to Early Childhood, Health, Mental Health, Medicine, Business, Criminal Justice, and Data Analytics, via 13 manuscripts and across 10 countries (USA and the UK to Chile, Norway, Spain, and Finland), offered thoughtful and calibrated directions for the future science and practice of school attendance. We invite your own careful reading of the papers, but here follow the main ideas:

## Ideas for change

1. Apply MD-Multi-tiered Systems of Support (MTSS) frameworks across geographical regions. While promising, these frameworks remain a work in progress. More data and innovative and sometimes radical reconfigurations at both systemic and analytic levels are sorely needed (Kearney and Graczyk).
  a. Conduct cross-discipline research using mixed methods allowing for (a) dissemination and use of broad findings and (b) establishment of common language and terminology (Heyne et al.).
2. Identify interventions to support children with chronic health conditions who participate in education. Begin with use of administrative data to address evidence gaps, compare interventions between authorities in schools and/or healthcare, and conduct randomized-controlled trials of interventions, developed with the input of children, young people, and their families (Jay et al.).
3. Further examine the differential effects of the pandemic on educational programs offering school administrators and policymakers ways to manage change (Korotchenko and Dobbs).
4. Validate models by incorporating additional external factors and exploring novel research questions about academic performance (Bowen et al.).
5. Use team science to discover innovative ways to promote attendance and prevent problematic absences. Outcomes could change with universal and targeted strategies and data platforms (Arbour et al.).
6. Dig deeper into underlying predictors of non-attendance within demographic groups to develop more effective interventions (Purtell and Ansari).
7. Compare racial and ethnic disparities in chronic absenteeism across school types or levels of intervention (LeBoeuf et al.).
8. Examine and record the effects of protective actions to prevent absenteeism and additive diagnoses and differences between adolescents with different combinations of neuroatypicalities (e.g., ADHD and autism spectrum disorder) (Niemi et al.).
9. Replicate studies on families with children with neurodevelopmental conditions who choose to de-register from school because of unmet needs (Paulauskaite et al.).
10. Employ new visions and theories of change brought on by advancements in human functioning (Kearney et al.).
11. Conduct studies on teachers' experiences after school re-openings, examining what did or did not work and why (Havik and Ingul).
12. Seek more inclusive paradigms for this postmodern era in favor of broad visions to fully unlearn calcified historical approaches (Kearney and Gonzálvez).

## Conclusions

Addressing the ideas for change requires removing old categorizations and processes and using modern approaches (Kearney and Gonzálvez), like machine learning, multi-tiered systems of support (Kearney and Graczyk), and data tracking to expose hidden factors influencing attendance. Using a tried but true cliché: it is a call to think outside the box!

When taken together, the findings from this Research Topic suggest we needed to recalibrate (Heyne et al.), especially after the COVID-19 pandemic (Havik and Ingul). We need assessment to shore up broken systems and support all students irrespective of how they accessed learning (e.g., homeschooling, special education, Montessori, or other), but we need to include teachers too (Havik and Ingul).

Unlearning has two parts. The first, “un”, is to undo, to stop, to pause. The second, the verb, learn. To gain, revise, or realign knowledge. We have tried to do both in this Research Topic of articles offering guidance for future exploration in the study of attendance and absenteeism.

As editors, we have brought to light the core of the work on school attendance and absenteeism and why it remains central. The numerous analyses presented here reflect the basic premise that schooling and attendance remain critical for belonging, emotional stability, and engagement in the learning process inside the school context. Missing school disrupts this continuity, leading to gaps. A void that is unreplaceable in any other environment.

## Author contributions

CG-G: Conceptualization, Project administration, Supervision, Writing—original draft, Writing—review & editing. AA: Writing—review & editing. MG: Writing—review & editing. IM: Writing—review & editing.
